# The Effects of Familial Social Support Relationships on Identity Meaning in Older Adults: A Longitudinal Investigation

**DOI:** 10.3389/fpsyg.2021.650051

**Published:** 2021-05-25

**Authors:** Aya Toyoshima, Jun Nakahara

**Affiliations:** ^1^Graduate School of Education, Kyoto University, Kyoto, Japan; ^2^Japan Society for the Promotion of Science, Research Fellowship for Young Scientists, Tokyo, Japan; ^3^School of Contemporary Sociology, Chukyo University, Nagoya, Japan

**Keywords:** social support, self-concept, symbolic interaction theory, family, older adults

## Abstract

This study aimed to examine whether social support promotes identity meaning among older adults. We hypothesized that when two spouses exchange social support, their sense of marital identity is enhanced. Among older adults, parental identity may be more strongly enhanced when parents provide social support to their children rather than receive social support from them. We conducted a longitudinal survey of 355 older adults (240 men and 115 women aged >60 years), who were assessed four times over 2 years. First, we confirmed the relationship between social support and identity meaning using an autoregressive path model. Second, we examined the effect of social support on the trajectory of role identities in a growth curve model. The intercepts of receiving support and providing support were significantly associated with the intercept of marital identity. In addition, the intercept of identity meaning for parents correlated with the intercept of providing support to their children but not with that of receiving support from their children. Social support between family members promotes role identities in family relationships. In particular, providing support to children correlates with parental roles which connect to subjective well-being.

## Introduction

Social support has long been considered an important factor in subjective well-being (Diener and Seligman, 2002). Geropsychological research on older adults has sought to identify factors related to well-being, such as aging-related changes in social roles. Some studies have found that subjective well-being is enhanced not only by receiving support (e.g., Matt and Dean, 1993; George, 2006), but also by providing support (Krause et al., 1992; Liang et al., 2001). When researchers examined the independent effects of each type of support, they found that the effects of social support depend on the relationship between the receiver and provider (Fukase and Okamoto, 2009; Thomas, 2010). For instance, Thomas (2010) reported that receiving support from a spouse was related to subjective well-being in older adults. However, receiving support from a child was related to a decrease in subjective well-being. Thomas (2010) also reported that providing support to a spouse was related to subjective well-being in older adults.

In this study, we examined the unique contributions of different sources of social support to identity meaning. To examine the relationships between receiving support, providing support, and identity meaning, we used a longitudinal dataset that enabled us to investigate reciprocal relationships in which individuals both receive and provide support.

### Theoretical Background

We examined the hypotheses associated with identity theory (Stryker and Burke, 2000). Identity meaning is a concept implicated in symbolic interaction theory (Blumer, 1986) and regarded as part of the structure that organizes self-concept. Individuals have multiple social roles and are able to engage in self-evaluation regarding individual roles (such as those of parent or grandparent). Identity meaning emerges from interactions between people who are connected to one another by specific social roles that influence behavior and emotion (Stryker and Burke, 2000).

Moreover, previous studies have verified the relationship between family interactions and identity meaning within a family (Reitzes and Mutran, 2006; Nakahara, 2011; Heintzelman and Bacon, 2015). Reitzes and Mutran (2004) demonstrated that positive identity meaning is related to subjective well-being, as also reported in a Japanese sample by Nakahara (2011) and Nakahara et al. (2012). Relying on support from others can diminish sense of competence in older adults (Siebert et al., 1999), and associated feelings of neediness and dependency can disturb identity meaning. These previous studies indicate that positive interactions between family members can enhance identity meaning in family members, which is connected to subjective well-being.

In this study, we focused on two kinds of familial relationships: marital relationships and parent-child relationships. These relationships are associated with strong social roles that guide how family members should interact. A stressor occurs in the lives of older adults when they enter retirement and lose the social role of worker (Gallo et al., 2006). Consequently, basic family roles such as parent and spouse, are likely to be more important in older adults than in younger individuals who are still in the workforce. Several previous studies have reported that both receiving and providing support were related to positive subjective well-being in spouses (e.g., Acitelli and Antonucci, 1994). However, the effects of social support in parent-child relationships are unclear (e.g., Thomas, 2010). Identity theory may be able to provide a framework for examining the distinct ways in which these different family roles contribute to subjective well-being.

### Social Support Among Spouses

Spouses are the main source of social support in older adults (Antonucci and Akiyama, 1987; Bookwala and Franks, 2005; Okabayashi and Hougham, 2014). Moreover, the level of social support between spouses has been found to predict extent of depressive symptoms (Choi and Ha, 2011) and subjective well-being (Acitelli and Antonucci, 1994).

Social support is likely to predict subjective evaluations regarding social interactions between individuals, and exchanges of social support between spouses can enhance an individual’s sense of identity as a spouse in a family. When older adults report that they receive support from their spouses, this generally indicates that they experience favorable social interactions with their spouses. Providing support to a spouse means allocating personal resources towards helping that individual. The amount of support provided by an older adult to their spouse can predict their subjective evaluation of their social interactions with their spouse. Thomas (2010) demonstrated that social support can be a valuable structural component of identity theory.

Support reciprocity is an important factor when relationships are examined in terms of receiving vs. providing support (Ingersoll-Dayton and Antonucci, 1988; Antonucci and Jackson, 1990; Buunk et al., 1993). Unbalanced exchanges of support, for instance, in which one person gives much more support than they receive, can be unpleasant (Antonucci and Jackson, 1990; La Gaipa, 1990). Ingersoll-Dayton and Antonucci (1988) reported that spousal relationships in which one spouse benefits more from the situation (i.e., provides less support than they receive) are associated with negative affect. Social support interactions between spouses are expected to be reciprocal, with the impact of receiving and providing support being equal. Indeed, support must be exchanged in both directions to maintain social support reciprocity.

### Social Support With Children

The support relationships between older adults and their children play an important role in maintaining or altering the social structure of the family. Many studies have examined the effects of social support from children on older adults’ subjective well-being (Cheng et al., 2013; Bangerter et al., 2015). These studies showed that both receiving and providing support between older parents and their children enhanced subjective well-being. However, receiving support from children had little effect on subjective well-being among older adults (Thomas, 2010). Silverstein et al. (1996) indicated that when parents receive more support from their children than they provide, this situation may violate the norms associated with their parental role.

The results of previous studies that tested the effects of providing support to children have not been consistent. For instance, Silverstein et al. (1996) and Thomas (2010) suggested that providing support produces a positive effect, whereas Takagi and Saito (2013) reported that they did not find a relationship between providing support and well-being. Antonucci and Jackson (1990) proposed the notion of the “support bank,” which relates to the exchange of support between a parent and child. Here, parent-to-child transfers are “longer term deposits [that] can be drawn on in future times of need” (Antonucci and Jackson, 1990, p. 179). Parents supporting their children buy in to a system of temporally generalized reciprocity without expecting an equivalent compensation. This way, the support balance between an older parent and their child does not have to be strictly controlled to maintain the subjective well-being of the older adult.

Our study hypotheses were guided by identity theory, which proposes that providing support to children enhances the identity meanings of parents. Thomas (2010) suggested that “if relationship norms are infringed upon, such as by receiving support from children when the norm throughout most of their lives is rather to provide support to them, then subjective well-being is likely to be lower.”

### Purpose and Hypothesis

First, these studies were cross-sectional. Thus, it is unclear whether the relationship between social support and identity meanings is demonstrated in a longitudinal study. Social support is related to identity meanings when using the framework of identity theory (Thomas, 2010; Heintzelman and Bacon, 2015). When the effects of both receiving and providing support are added to a model separately, it is difficult to examine which support is more effective. This is because participants who report a high level of received support tend to report a high level of provided support. Second, whether the nature of the social support relationship (with spouse or child) influences the effect on identity meaning is still unclear. We hypothesized that social support affects positive role identity.

To summarize, we aimed to examine whether social support predicts identity meaning, and to assess differences associated with the source of support in the relationship. To identify relationships between receiving support, providing support, and identity meaning, we used a longitudinal dataset. We aimed to examine three hypotheses.

Hypothesis 1: Receiving and providing support predict positive identity meaning in older adults.
Hypothesis 2: In exchanging support with a spouse, both receiving and providing support predict identity meaning as a spouse.
Hypothesis 3: In exchanging support with children, providing support predicts identity meaning as a parent more so than receiving support.

## Materials and Methods

### Participants

We conducted a four-wave longitudinal survey over the course of 2 years. Participants were Japanese people aged 60 and older who lived in the Osaka prefecture. They were assessed four times over 2 years (First assessment: January–February 2012; Second: September–November 2012; Third: May–June 2013; and Fourth: February–March 2014). In January 2012, we recruited older adults at the Silver Human Resource Center in Osaka prefecture, which is a platform for older people to find employment. The Silver Human Resource Center sent questionnaires to those participants who responded to the advertisement. The participants who completed and returned the questionnaire to the Center were financially compensated (approximately 10 United States dollars) for completing each assessment.

In total, 279 participants had answered the questionnaire at T1, but 67 had dropped out by T4. To cover the sample size, 29 participants were added in T2, 36 participants in T3, and 11 participants in T4. The final sample included 355 older adults (240 men and 115 women). Forty participants answered that they did not have spouse (3 divorced, 31 widowed, and 6 never married), and 24 participants reported that they did not have any children. The analysis data for marital relationships comprised responses from 265 participants and excluded participants who did not have spouse or had missing values for all variables. The analysis data for parental relationships comprised responses from 281 participants and excluded participants who did not have children or had missing values for all variables.

In this study, we estimated missing values using the full information maximum likelihood (FIML) method. This study was conducted at the Silver Human Resource Center for older people, and almost all participants were homogeneous in terms of health and economic status. We estimated that the missing values occurred randomly and, by using FIML, had the benefit of maintaining the sample size for analysis.

### Measures

The survey was composed of four main parts, which contained items associated with role identity, social support, subjective well-being, and background variables.

#### Identity Meanings as Spouse and Parent

We used the measurement of identity meanings developed by Reitzes and Mutran (2002). The scale was developed to assess satisfaction with particular social roles that reflect role identity meaning. For instance, to measure identity meaning as a spouse, respondents completed a phrase that began “As a husband/wife, I am…” by choosing responses to 10 adjective pairs organized in a semantic differential five-point format (Osgood et al., 1957). We used the following adjective items: “A. active–B. inactive,” “A. successful–B. unsuccessful,” “A. competent–B. not competent,” “A. relaxed–B. tense,” “A. happy–B. sad,” “A. confident–B. not confident,” “A. warm–B. cold,” “A. open–B. closed,” “A. interested in others–B. interested in self,” and “A. sociable–B. solitary.” The response options were “1. A,” “2. Almost A,” “3. Neither,” “4. Almost B,” “5. B”. To measure identity meaning as a parent, we used the following items: “As a parent, I am…”. We used the total scores of 10 items for the calculations, with a higher score indicating that the identity meaning wass positive. In the current sample, we calculated Cronbach’s α using the first wave dataset. The values for spouse (Cronbach’s α = 0.89) and parent (Cronbach’s α = 0.92) indicated an appropriate level of internal consistency.

#### Social Support

We assessed the social support relationships between the spouses and children of the participants using the measurement developed by Sugisawa et al. (1994). This scale is used to measure participant perceptions of the support they received from others. The scale includes two items of emotional support (“How does your spouse/child talk to you about your concerns when you are feeling uncertain about something?” and “How does your spouse/child show consideration for your feelings?”) and an instrumental support item (“How does your spouse/child make sure that they care for you when you are ill?”). Each item on the scale was rated on a five-point Likert scale ranging from 1 = “Not at all” to 5 = “Very well.” To measure the provision of support from the participant to their spouse or child, we changed the wording of the items accordingly (“How do you talk your spouse/child about their concerns, when he or she is feeling uncertain about something?”; “How do you show consideration for your spouse/child?”; and “How do you make sure that you care for your spouse/child if he or she is ill?”). We used the total scores of three items of support for calculations, with a higher score meaning that the person reported higher support. In the current sample, all items related to receiving and providing support displayed an appropriate level of internal consistency (receiving support from spouse: α = 0.83; receiving support from child: α = 0.77; providing support to spouse: α = 82; and providing support to child: = 0.84).

#### Background Variables

The survey items included age, gender, subjective health, subjective economic condition, education, marital status, and child status. Participants rated items utilizing a Likert scale. Subjective health was rated from 1 (extremely poor) to 5 (extremely good), and subjective economic condition was rated from 1 (extremely poor) to 5 (extremely rich). The participants indicated their education status as 1 (elementary school graduate, 1–6 years), 2 (junior high school graduate, 7–9 years), 3 (high school graduate, 10–12 years), 4 (college graduate, 13–16 years), or 5 (entered graduate school, >17 years). The participants indicated their marital status as 1 (living together), 2 (separated), 3 (divorced), 4 (bereaved), or 5 (never married). For child status, participants chose one of the three categories: 1 (living with their children), 2 (not living with their children), or 3 (no children).

### Data Analysis

Analyses were performed using SPSS 19.0 for Windows (IBM Inc., Chicago, IL, United States) and M plus7 (Muthén and Muthén, Los Angeles, CA, United States). To assess goodness-of-fit, we used the criteria that the CFI > 0.9 (Bentler and Bonnet, 1980) and RMSEA < 0.10 (Browne and Cudeck, 1993).

**FIGURE 1:**
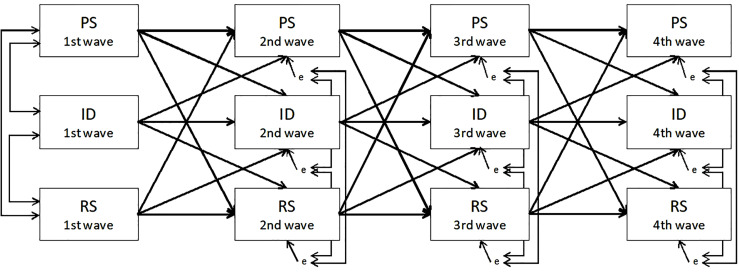
The basic analytical model for autoregressive path model. PS, providing support; RS, receiving support; ID, identity meanings.

Hypothesis 1 was examined using an autoregressive path model with a structural equation model (Lazarsfeld and Fiske, 1938; Finkel, 1995). This design involves examining whether the variables in primer predict the later variables over time. We used an analytical model in which three variables (identity meanings, receiving support, and providing support) were modeled with an autoregressive structure, simultaneously assessed variables were tested for correlations, and each variable was predicted by previous variables (Figure 1). The model includes the implication of a bidirectional relationship between the variables. Before examining individual transition over time (Hypotheses 2 and 3), we tested Hypothesis 1 to confirm whether receiving and providing support predict the identity meaning at a later wave. The paths from supports to identity meaning (Figure 1) indicate that social support predicts the score on the other measure at a later wave, and the paths from identity meaning indicate a relation of in the opposite direction. The relationships between variables indicate the prospective effect of one variable on the other (e.g., the effect of identity meaning in the 1st wave on identity meaning in the 2nd wave), after controlling for stability across time (e.g., the residual of identity meaning in the 2nd wave and the residual of receiving support in the 2nd wave). The stability and coefficients were constrained such that they were equal across time.

**FIGURE 2:**
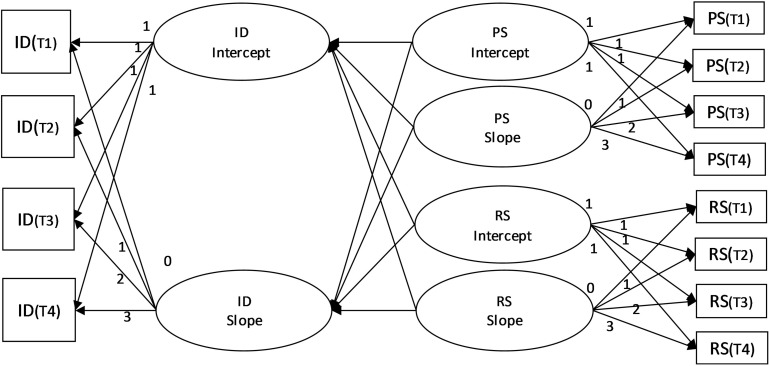
The analytical model for the latent growth model. PS, providing support; RS, receiving support; ID, identity meanings.

Hypotheses 2 and 3 were examined using a latent growth model, which is used to examine variables as predictors of transition over time (McArdle, 2009; McArdle et al., 2009). To examine the effects of receiving and providing support on identity meaning, the analytical model assumed the slopes and intercepts of receiving support and providing support predicted those of identity meaning (Figure 2). The intercepts indicate the basic score of variables at T1, and the slopes indicate variation of the score between T1 and T4. If the intercept of identity meaning is positively associated with the slope of support, it means the scores of supports increased between T1 and T4 among participants who rated positive identity in T1. If the slope of providing support positively affects the slope of identity meanings, it means that the increased provision of support to the family was associated with an increased score of identity meaning.

## Results

Table 1 presents the participant demographics for background variables. The mean age was 69.27 years (SD = 4.3) at T1, and 67.61% of participants were men. Responses indicated that 60% of the participants had graduated from high school. Table 2 summarizes the descriptive statistics of identity meanings and social support.

**TABLE 1:** Descriptive statistics.

		*N*	%
Gender	Male	240	67.61
	Female	115	32.39
Subjective health	Extremely poor	0	−
	Poor	21	5.92
	Neither	45	12.68
	Good	251	70.70
	Extremely good	38	10.70
Subjective economic condition	Extremely poor	8	2.25
	Poor	101	28.45
	Neither	213	60.00
	Rich	27	7.61
	Extremely rich	4	1.13
	No answer	2	0.60
Past schooling	Elementary school graduate	8	2.25
	Junior high school graduate	101	28.45
	High school graduate	213	60.00
	College graduate	27	7.61
	Entered graduate school	4	1.13
Marital status	Living together	296	83.38
	Separated	4	1.13
	Divorced	17	4.79
	Widowed	31	8.73
	Never been married	6	1.69
	No answer	1	0.30
Child status	Living with children	132	37.18
	Not living with children	198	55.77
	No children	24	6.76
	No answer	1	0.30

**TABLE 2:** Descriptive statistics of identity meanings and social support.

	Marital	Parental
	M (SD)	M (SD)
**Identity meanings**		
T1	3.65 (0.62)	3.64 (0.68)
T2	3.55 (0.69)	3.64 (0.61)
T3	3.60 (0.64)	3.66 (0.64)
T4	3.56 (0.64)	3.68 (0.68)
**Providing support**		
T1	3.73 (0.72)	3.72 (0.71)
T2	3.69 (0.67)	3.70 (0.69)
T3	3.71 (0.65)	3.71 (0.71)
T4	3.72 (0.63)	3.67 (0.74)
**Receiving support**		
T1	3.69 (0.77)	3.45 (0.69)
T2	3.65 (0.75)	3.43 (0.69)
T3	3.64 (0.78)	3.45 (0.73)
T4	3.67 (0.79)	3.43 (0.74)

### The Effects of Social Support on Identity Meanings

Before we examined the effects of social support on identity meanings, we used autoregressive path models to test whether receiving support and providing support predict identity meanings (Figure 1) for marital and parent–child relationships. The goodness-of-fit values for both models were appropriate (marital relationship: χ2 (45) = 215.23, *p* < 0.001, RMSEA = 0.11, CFI = 0.90; parent–child relationship: χ2 (45) = 162.51, *p* < 0.001; RMSEA = 0.09, CFI = 0.93). Table 3 shows the estimates for the stability and path coefficients in the models of marital and parent–child relationships.

**TABLE 3:** Results of the autoregressive path model.

	Marital relationship	Parent–child relationship
ID→ID	0.47**	0.53**
RS→RS	0.75**	0.60**
PS→PS	0.63**	0.61**
ID→RS	0.07*	0.11**
RS→ID	0.14**	0.09**
ID→PS	0.10**	0.11**
PS→ID	0.14**	0.17**
PS→RS	0.02	0.06*
RS→PS	0.06	0.04

The values are standardized beta, which represent the effect of the path. PS, providing support; ID, identity meanings; RS, receiving support, **p < 0.01, *p < 0.05.

In both models, all paths between identity meaning and support were significant. However, the paths from receiving support to providing support were not significant in the marital relationship model, and the coefficient of the path from providing support to receiving support in the parent–child model just barely reached significance.

### Differential Effect of Receiving vs. Providing Support on Identity Meanings

We next used growth curve models (Figure 2) to examine the effects of receiving vs. providing support on the trajectories of identity meaning. Table 4 shows the results of the unconditional model and the standardized path coefficients for both models. The goodness-of-fit values in the marital model, which assumed that social support between spouses would predict identity meaning in individual spouses, were appropriate [χ2 (df = 39) = 50.72, n.s.; RMSEA = 0.03; CFI = 0.99]. In the hypothesis model, the intercepts of receiving support (β = 0.30, *p* < 0.05) and providing support (β = 0.38, *p* < 0.01) were significantly associated with the intercept of identity meaning as a spouse. However, the paths from the slopes of both providing and receiving support to the slope of identity meaning as a spouse were not significant. The goodness-of-fit values in the parental model, which assumed that social support between spouses would predict identity meaning in parents, were appropriate [χ2 (df = 39) = 27.03, n.s.; RMSEA = 0.00; CFI = 1.00]. The intercept of providing support was significantly associated with the intercept of identity meaning as a parent (β = 0.69, *p* < 0.01), although the path from the intercept of receiving support to the intercept of identity meaning as a parent was not significant (β = 0.14, n.s.). The paths from the slopes of both providing and receiving support to the slope of identity meaning as a parent were not significant.

**TABLE 4:** Results of the latent growth model.

		Marital relationship	Parent-child relationship
**Unconditional model**			
ID	Intercept parameter	3.63**	3.62**
	Slope parameter	−0.03*	0.01
	Intercept variance	0.25**	0.23**
	Slope variance	0.01	0.00
RS	Intercept parameter	3.71**	3.46*
	Slope parameter	–0.02	–0.01
	Intercept variance	0.46**	0.34**
	Slope variance	0.01	0.02**
PS	Intercept parameter	3.71**	3.71**
	Slope parameter	0.00	–0.01
	Intercept variance	0.35**	0.34**
	Slope variance	0.01	0.01*
**Hypothesis model**			
PS Intercept → ID Intercept		0.38**	0.69**
PS Intercept → ID Slope		0.14	–0.17
PS Slope → ID Intercept		–0.19	–0.01
PS Slope → ID Slope		0.83	0.62
RS Intercept → ID Intercept		0.30*	0.14
RS Intercept → ID Slope		0.36	0.23
RS Slope → ID Intercept		0.03	0.11
RS Slope → ID Slope		0.16	0.26

The values of Unconditional model are unstandardized. The values of Hypothesis model are standardized beta, which represent the effects of the path between intercepts of slopes of the variables. PS, providing support; ID, identity meanings; RS, receiving support, **p < 0.01, *p < 0.05.

## Discussion

Guided by identity theory, we examined whether social support enhances identity meaning and tested the contributions of different sources of support by considering marital and parent–child relationships. Our results indicate that both receiving and providing support were related to identity meaning, although the causal relationship between these two variables is still unclear. Both the marital and parental models revealed bidirectional relationships between social support and identity meaning. For the growth curve model, the intercepts of social support predicted the intercepts of identity meanings. This means that the participants who frequently exchanged social support were more likely to evaluate their social role as positive. Thus, exchanging social support was related to identity meaning in terms of the relationship between the receiver and provider, as predicted by the identity theory. Providing support to others promotes subjective well-being in older adults by boosting their identity meaning with respect to a specific social role (Thomas, 2010). The results of this study suggest that exchanging social support with a spouse or child enhances the level of satisfaction with the social role of spouse or parent, and it is thus connected to the maintenance of subjective well-being.

Although the growth curve model indicated that receiving or providing support predicted identity meanings, we were not able to confirm a specific direction of effect according to which social support enhanced identity meaning. Hence, our first hypothesis was not supported. Identity meaning as a spouse and/or parent may be stable across time, and thus may be unlikely to change during a 2-year period among a group of older adults. For older adults, social interactions with their spouse and children are likely to have been relatively consistent from the point at which the children reached adolescence. Indeed, such interactions are not likely to undergo a short-term transformation. However, older adults with grandchildren may show different patterns (Reitzes and Mutran, 2004; Nakahara, 2011; Nakahara et al., 2012).

Providing support to children was related to the amount of support that the participants received; however, receiving support from children was not related to providing support. The results of the autoregressive path model showed this effect for parent–child relationships but not marital relationships. When older adults provided support to their children, they received support from their children in a way that supports the idea of a “support bank” (Antonucci and Jackson, 1990). However, we did not observe the reverse of this relationship: receipt of support did not lead to increased provision of support in either marital or parental relationships. Thus, we did not observe reciprocity in support exchange, with receipt of support relating to the subsequent provision of support.

We analyzed the longitudinal data using the growth curve model and tested Hypotheses 2 and 3. Our results indicated that both providing support and receiving support influenced identity meaning as a spouse, thus supporting Hypothesis 2. The role of spouse is associated with clear social norms, that is, spouses are expected to help one another, and the support received can lead to enhanced subjective well-being (Thomas, 2010). The social role of spouse often carries the expectation of an equal relationship, such that unbalanced exchanges of support with a spouse can lead to unpleasant feelings (Antonucci and Jackson, 1990; La Gaipa, 1990). Thus, it is logical that both receiving support from a spouse and providing support to a spouse are important factors in identity meaning as a spouse.

For older adults, providing support to their children was related to identity meaning, although receiving support from their children did not have the same relationship. These results supported our third hypothesis. Older parents who reported that they had provided support to their children appeared to develop a stronger identity as a parent and were more satisfied with their social role. However, receiving support from their children was not related to identity meaning as a parent. When older parents receive increasing amounts of support from their children, this can conflict with social parental roles (Silverstein et al., 1996). Thus, it is important for older adults to provide support to their children to maintain their parental role.

Several limitations of this study should be noted. First, Japan has a specific cultural code related to family support for older adults, termed filial piety (Takagi and Saito, 2013), and familial roles can be very culturally specific; thus revalidation in other Western and Asian countries will be necessary. Second, the measurements used in this study had limitations concerning reliability and validity. The alphas for the measurement of social support were under 0.70 and included three items. This is because these measures summarized multiple types of support: emotional support and instrumental support. In this study, it remains unclear what kinds of support are effective. As a next step, the effects of different kinds of support (e.g., emotional or instrumental, whether support was needed by recipients) should be examined separately using an appropriate scale. Moreover, social support was measured using self-rated variables, and it is not clear that these scales reflected actual social exchange and transmission of support. Finally, our sample was local. All respondents were recruited at one location, the Silver Human Resource Center for older people in Osaka prefecture. The respondents were biased in that the ratio of men was higher than the average for people aged 65 or older in Japan (43%; Statistics Bureau of Japan, 2016). A possible reason may be that more men than women are registered at the center. A larger sample from other facilities and areas would enable us to explore differences by gender and social background.

## Conclusion

The results of our study using the longitudinal data indicated that both receiving support from a spouse and providing support to a spouse related to identity meaning as a spouse, while only provision of support to children was related to identity meaning as a parent. In this study, we did not find longitudinal changes of social support and identity meanings, because identity meaning as a spouse and/or parent may be stable across time among older adults.

## Data Availability Statement

The raw data supporting the conclusions of this article will be made available by the authors, without undue reservation.

## Ethics Statement

The studies involving human participants were reviewed and approved by Osaka University Graduate School of Human Sciences Research Ethics Committee. The patients/participants provided their written informed consent to participate in this study.

## Author Contributions

AT: conceptualization, software, validation, formal analysis, investigation, data curation, writing – original draft, writing – review and editing, and visualization. JN: conceptualization, methodology, investigation, writing – review and editing, supervision, and project administration. Both authors contributed to the article and approved the submitted version.

## Conflict of Interest

The authors declare that the research was conducted in the absence of any commercial or financial relationships that could be construed as a potential conflict of interest.
